# Beer Safety: New Challenges and Future Trends within Craft and Large-Scale Production

**DOI:** 10.3390/foods11172693

**Published:** 2022-09-03

**Authors:** Călina Ciont, Alexandra Epuran, Andreea Diana Kerezsi, Teodora Emilia Coldea, Elena Mudura, Antonella Pasqualone, Haifeng Zhao, Ramona Suharoschi, Frank Vriesekoop, Oana Lelia Pop

**Affiliations:** 1 Department of Food Science, University of Agricultural Sciences and Veterinary Medicine, 400372 Cluj-Napoca, Romania; 2 Molecular Nutrition and Proteomics Lab, CDS3, Life Science Institute, University of Agricultural Science and Veterinary Medicine, 400372 Cluj-Napoca, Romania; 3 Gembloux Agro-Bio Tech, Department of Food Science and Formulation, University of Liège, B-5030 Gembloux, Belgium; 4 Department of Food Engineering, University of Agricultural Sciences and Veterinary Medicine, 400372 Cluj-Napoca, Romania; 5 Department of Soil, Plant and Food Science (DISSPA), University of Bari Aldo Moro, I-70126 Bari, Italy; 6 School of Food Science and Engineering, South China University of Technology, Guangzhou 510640, China; 7 Department of Food Technology and Innovation, Harper Adams University, Newport TF10 8NB, UK

**Keywords:** beer, contamination, microbiological spoilage, chemical contaminants

## Abstract

The presence of physical, chemical, or microbiological contaminants in beer represents a broad and worthy problem with potential implications for human health. The expansion of beer types makes it more and more appreciated for the sensorial properties and health benefits of fermentation and functional ingredients, leading to significant consumed quantities. Contaminant sources are the raw materials, risks that may occur in the production processes (poor sanitation, incorrect pasteurisation), the factory environment (air pollution), or inadequate (ethanol) consumption. We evaluated the presence of these contaminants in different beer types. This review covers publications that discuss the presence of bacteria (*Lactobacillus*, *Pediococcus*), yeasts (*Saccharomyces*, *Candida*), moulds (*Fusarium*, *Aspergillus*), mycotoxins, heavy metals, biogenic amines, and micro- and nano-plastic in beer products, ending with a discussion regarding the identified gaps in current risk reduction or elimination strategies.

## 1. Introduction

Beer is often seen as a product of high repute and traditional craftsmanship, produced with ingredients that have been a staple for all layers of society dating back to Neolithic times and the Mesopotamians around 6000–7000 years ago [1]. Despite the obvious presence of alcohol, which brings about the often-experienced feelings of euphoria when alcohol is consumed in moderation, beer has been consumed in preference over town water due to its inherent safety, which is linked to the numerous antimicrobial hurdles associated with beer. As such, beer has gained a reputation as a staple beverage with a solid and trustworthy authenticity. However, like all manufactured products, even beer is not immune from exposure to intrinsic and extrinsic contaminants [1,2].

Chemical contamination can occur from cultivation, processing, and packaging. For example, crop contaminations with mycotoxins (nivalenol (NIV), deoxynivalenol (DON), zearalenone (ZEA), deoxynivalenol-3-glucoside (DON-3-Glc), fusarenon-X (FUS-X), 3-acetyl-deoxynivalenol (3-ADON), 15-acetyl-deoxynivalenol (15-ADON), HT-2 toxin (HT-2), and T-2 toxin (T-2)) are estimated to be between 60 and 80% with 45% more than 40 years ago [3]. Estimating human exposure to micro-and nano-plastics and biogenic amines is pivotal for scientists and authorities responsible for public health [4,5]. 

Microbial and fungal contamination (*Pectinatus*, *Megasphaera*, *Staphylococcus*, *Bacillus*, *Enterobacter*, and *Zymomonas*) of beer are not well-documented but are not neglected due to important health implications [6]. 

Being aware of the possible presence of different sorts of contaminants in foods, in this case, beer, may help apply different strategies to increase the control and diminish the harmful effects. In this review, we want to highlight beer’s most common contaminants and the ones with a higher incidence and significant impact on product processing and human health. Fermented cereals high in sugars generated from starches are ideal for preparing the alcoholic beverage known as beer. Ale beer is typically fermented by top-fermenting yeast, *Saccharomyces cerevisiae*, while lager beer is generally fermented by bottom-fermenting strains of *Saccharomyces pastorianus*, a hybrid yeast, and is more like the beer prepared in ancient times [7]. Fermented beverages, such as beer, have multiple health benefits contributing to minerals, vitamins, polyphenols, and fibres [8].

Because ethanol is present (0.5–10% *w*/*w*), as well as, hop bitter compounds (approximately 17–55 ppm of iso-α-acids), low pH (3.8–4.7), and high carbon dioxide content (approximately 0.5% *w*/*v*), beer is a microbiologically stable beverage [9]. 

Generally speaking, there are four basic stages in the brewing process: the preparation of the wort, fermentation, maturation, and filtration and/or stabilisation [10]. Before the brewing process, barley is subjected to malting, a process that permits the maturation of enzymes. Furthermore, the enzymes are able to break down the complex starches in the grain into simple fermentable sugars. These sugars will eventually be employed in the mashing process. Malted barley represents the most important cereal used in the brewing process, followed by wheat, corn, wheat malt, rice, and millet. The grains are first milled and then transferred to a saccharification kettle, where, with the addition of water, mash is obtained. Usually, saccharification takes up to two hours, depending on the mashing diagram. Then, the mash is filtered to separate the brewer’s spent grain, the solid portions, and wort, the liquid portion that will be further boiled.

Hops or hop-derived products, such as hop pellets or essential oils, are added during the boiling process. Hops have a distinct flavour and aroma that have a big impact on the finished product. Thus, the dosage needs to be tailored to the intended beer’s profile and harmoniously incorporated into the matrix as the beer matures.

After the boil, trub (or coagulated proteins) and suspended hop particles (brewer’s spent hops) are present in the wort that must be removed, frequently using a “whirlpool tank” or “settling tank.” The clear wort is then pumped into the fermentation tanks, where yeasts are introduced once it has been cooled and aerated. During the fermentation process, yeasts consume sugars and amino acids from the wort. Under anaerobic conditions, the sugars are digested and primarily transformed into ethanol and carbon dioxide. Aldehydes, ketones, higher alcohols, organic acids, and esters are only a few of the volatile metabolites that alcoholic fermentation produces, which are referred to as “fermentation by-products” or “congeners.” The main determinants of the beer’s quality are fermenting time and temperature. Ale beers ferment at 16–18 °C, while lager beers ferment at 7–14 °C. 

Fermentation slows after all the fermentable sugars have been consumed. Beer can subsequently be separated from the yeast biomass through a procedure called racking. Just-produced, or “green,” beer can then move on to the latter steps of processing, which include maturation, filtration, and packaging. The temperature is kept at or below 0 °C during this time, avoiding oxygen contact. 

When the beer finishes maturing, it is pumped to the filtration room. Here, beer is passed through filters coated with diatomaceous earth to remove suspended particles and unhinge potential turbidity producers (stabilisation). This procedure is crucial to the preservation of the beer so that there are no long-term observable changes and the beer retains its original appearance. 

Finally, beer is pasteurised and packaged in bottles, cans, or kegs to maintain the product’s flavour until it is consumed.

Every type of beer is different from each other by its specific properties, such as colour, alcohol content, clarity, flavour, bitterness, ingredients, and even microbial diversity. Later in this review are presented the ways that water, the other ingredients, and the brewing process might contribute to beer contamination and which are the most intensively reported contaminated beers. Despite the fact that craft beer constantly increases the consumers interest, the awareness of safety issues also arises among the craft brewers. The beer technologies already implemented to reduce beer contamination are also discussed (i.e., fermentation) [11].

## 2. Beer Safety

Due to the increased demand for beer, it is necessary to investigate and pay attention to possible contaminants that could affect consumers’ health. Even if beer is not an excellent medium for organisms to grow, some species of microorganisms can grow in beer, changing its properties and causing turbidity and off-flavours [12]. Craft beers are more prone to spoilage than beer prepared in large-scale breweries [13], probably because they are less likely to be pasteurised or sterile-filtered. The reported contamination agents in beer are presented in Table 1. 

Beer spoilage causes economic losses to breweries and even the loss of consumers’ confidence. Beer-spoilage bacteria are part of Gram-positive lactic acid bacteria and Gram-negative acetic acid bacteria [18,19,20,21,22]. The other microorganisms that can cause beer spoil are not well-documented and their properties to contaminate beer need to be studied. Spoilage and contamination of beer in rare cases were produced by bacteria belonging to *Staphylococcus*, *Bacillus*, *Enterobacter*, and *Zymomonas* genera, changing the pH of the final product, creating sediment, ropiness, haze, and off-flavour [23]. One of the most critical factors in cell growth in beer is temperature. With decreasing the temperature from 35 °C to 4 °C, beer spoilage decreased significantly, meaning low temperature plays an essential role in protecting beer from spoilage, for example, with *Staphylococcus xylosus* [24]. Beer quality can be compromised by mycotoxins produced by fungal contamination of malting barley [25,26]. Regulations on the maximum tolerable level of deoxynivalenol, for example, differ from country to country and may vary from 0.75 µg/g to 1.17 µg/g barley [24]. Physical, chemical, and biological treatments are used during the malting process to decrease fungal contamination. One way to reduce fungal contamination is using microorganisms as antifungal treatment, which is desirable because they exhibit environmental sensitivity and sustainability [27]. However, their efficiency can be affected by microbial interactions or different side effects. A study describes a more effective bio-fungicide used in the malting process, using Reverse transcription - Polymerase Chain Reaction (RT-PCR) to quantify the antifungal potential of oomycetes *Pythium oligandrum* on barley naturally or artificially contaminated with three *Fusarium* species [28].

Biogenic amines represent a significant group of chemical contaminants in beer. In a paper, 118 samples of craft beer manufactured in microbreweries of Central Europe were analysed. The results showed that more than 30% of the samples had a total biogenic amines content between 50–100 mg/L. The most frequently detected biogenic amines were tyramine, putrescine, and cadaverine. However, 18% of the craft beer samples had a total amount of biogenic amines higher than 100 mg/L [29].

**Figure 1:**
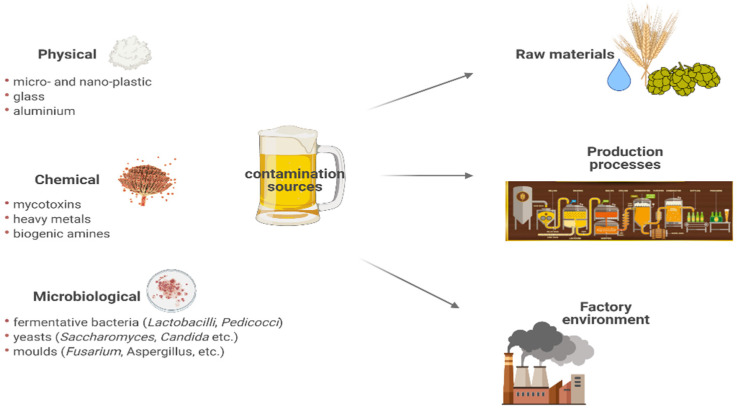
Beer contamination sources.

Due to its extensive consumption, beer contamination with mycotoxins needs to be limited as a priority for consumers’ health. The beer technology includes operations that can increase or decrease the initial level of mycotoxins. It was reported that mashing might decrease the level of mycotoxins—ochratoxin A (OTA), aflatoxin B2 (AFB2), fumonisin B2 (FMB2), aflatoxin G1 (AFG1), aflatoxin B1 (AFB1), zearalenone (ZON), and patulin (PAT)—by 50% from their initial level [30]. Other studies showed that fermentation could completely remove certain mycotoxins (i.e., ZON and PAT) [11,31,32]. The elimination of the mycotoxins from the beer is mostly dependent on their absorption to the spent grains. The most critical beer production processes positively impact reducing mycotoxin levels: steeping, kilning, mashing, fermentation, and clarification [26]. In common, concentrations of biogenic amines up to 100 mg/kg or 100 mg/L are considered to be safe for the customer. In any case, different compounds, such as ethanol and different drugs, can essentially diminish the body capacity to transform these compounds [28]. Figure 1 shows the beer contamination sources. 

## 3. Microbiological Safety of Beer

It is often more challenging to attain the same exacting hygiene procedures when brewing at a smaller scale [13]. Conventional and mostly unconventional raw materials, such as fruits, herbs, honey, spices, and vegetables, added post-wort boil increase the risk of beer microbial spoilage because of their own microbial load [9,33]. A general founded impression is that pathogenic microorganisms’ survival in beer is low due to various inhibitory factors such as ethanol (0.5–10% (*w*/*w*)), hop-bittering compounds, low pH (3.8–4.7), carbon dioxide, low oxygen, and the lack of nutritional substrates [31,34], factors derived from the technological flow of beer production. Nevertheless, the current trend in beer production (lower ethanol and bitterness) might pose a potential risk of beer spoilage [13,32]. There are a few exceptions of Gram-positive bacteria, such as *Lactobacillus* and *Pediococcus*, species that can grow in beer [35]. For the lactic acid bacteria, resistance to hop is crucial for their ability to survive and grow in beer. Hop compounds, mainly iso-α-acids in beer, have antibacterial activity against Gram-positive bacteria [32].

**Figure 2:**
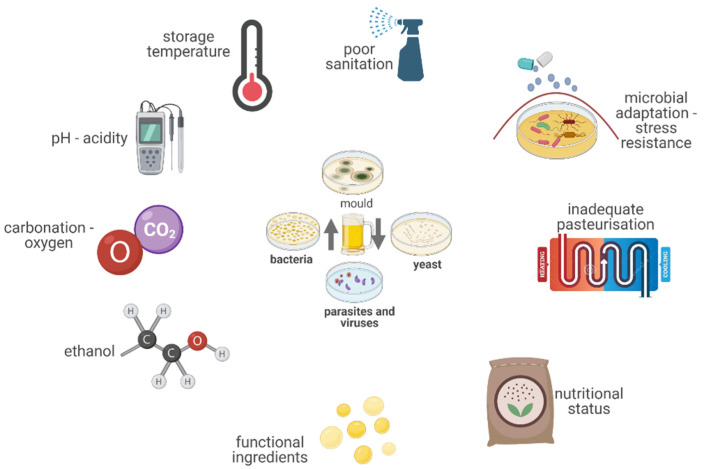
Microbial spoilage of beer.

Microbiological contaminants can spoil beer from various sources (Figure 2). Primary contaminants are derived from the raw materials and the brewing equipment, while secondary contaminants are introduced to the finished product during the bottling, canning, or kegging. Approximately 50% of the documented microbiological contaminations can be attributed to secondary contaminations, but the primary contamination is more harmful because it can compromise a complete brew [36]. The contamination of beer via the brewing equipment is caused by improper cleaning and sanitation procedures [36]. 

### 3.1. Lactic Acid Bacteria and other Fermentative Bacteria and Beer Spoilage

Beer has a low pH (3.8–4.7) and, due to yeast fermentation, has a selective nutritional concentration that is insufficient for the development of many bacteria [31].

Despite these unfavourable characteristics, a small number of microorganisms are capable of spoiling the beer. Beer-spoiling bacteria constitute a significant problem for the brewing business worldwide, as spoilage incidences can harm brand equity and cause expensive product retrieval costs [37].

Various unpleasant sensory alterations are frequent indicators of microbial infection. A strain of *Staphylococcus xylosus* has been reported in commercial turbid and off-flavoured craft beer pulled from the local market by breweries. *S. xylosus* is a microbe that lives on the skin of people and animals, as well as a common bacteria found in food and raw materials [38]. This strain grew well in the presence of hop chemicals and had a high potential to ruin beer.

Beer’s sour flavour is frequently linked to acetic and lactic acid bacteria [39]. The two lactic acid bacteria that are thought to be most widespread in beer are *P. damnosus* and *Lb. brevis*. Strong buttery and diacetyl off-flavours are typically signs of *P. damnosus* infection. Less frequent contaminations are caused by *Lb. lindneri* and *Lb. plantarum* [13].

As shown in Table 2, LAB are the most common bacteria that cause beer deterioration. Deng et al. mention that LAB accounts for 60–90% of microbiological hazards in breweries. Typical LAB degradation of beer results in turbidity, acidity, gas production, and off-flavours due to the creation of side metabolites. *Lactobacillus brevis, Lactobacillus lindneri*, and *Pediococcus damnosus* are the most commonly isolated beer-spoilage LAB [37]. 

### 3.2. Yeasts and Beer Spoilage

Yeasts are chemoorganotrophic microfungi that get their carbon and energy from digesting organic matter. Before the boiling process, processes in beer technology represent the lowest risk of yeast contamination because yeasts are not thermotolerant and cannot survive even the slightest deviation of the boiling process. Filtration is the current technology for the removal of yeast from beer. *Saccharomyces cerevisiae* and *Pichia kudriavzevii* residue have shown to be the most resistant to gastrointestinal conditions in vitro, suggesting that the wastes obtained from brewery would become a high-value probiotic product [43]. Traditional growth-based procedures and modern molecular technologies can be used to detect beer-spoilage yeast [41]. The use of dual magnetic/light-responsive self-propelled microrobots has proven to be an effective method for capturing yeast cells, thus reducing the significant amount of spoilage yeasts [36]. Polymerase chain reaction (PCR) was helpful for the simultaneous detection and identification of *B. custersianus, D. bruxellensis, D. anomala*, and other brewer’s yeast species [41]. In addition to molecular techniques such as PCR and rRNA hybridisation, the applicability of molecular profiling using matrix-assisted laser desorption/ionisation time-of-flight mass spectrometry (MALDI-TOF MS) in combination with Biotyper software was investigated with good results and remarkable advantages (cost- and time-effective) [18].

The most frequent indicators of a spoiled beer by yeast are the generation of surface film, off-flavour, and turbidity owing to wild yeast’s non-flocculent potentials [44]. Due to the increasing popularity of craft beers and non-traditional products in the brewing industry, wild yeasts are becoming increasingly important. Non-alcoholic and low-alcoholic flavoured beers are microbiologically unstable due to their high sugar content, which can increase the number of spoiled wild yeast species [45]. *Saccharomyces, Pichia, Rhodotorula, Alternaria, Hansenia, Wickerhamomyces,* and *Cladosporium* were identified in non-alcoholic beer before pasteurisation [46]. To investigate beer spoiling capabilities, *Dekkera/Brettanomyces* yeast was inoculated into two commercial bottled beers (Japanese pilsner-type and malt beer) at a concentration of 10^3^ cells/mL (10^5^ cells/bottle). The specie *Brettanomyces custersianus* was associated with turbidity in beer [41]. The *Trigonopsis cantarellii* strain has been reported to produce a substantially higher amount of trans geraniol monoterpene alcohol and unfavourable aromas [40]. Two strains of wild yeast, *T. cantarellii* and *Candida sojae*, produced beers with organoleptic properties similar to a commercial lager beer, thus suggesting that these beer contaminants could be repurposed for beneficial use in beer fermentation, with a particular focus on low-alcohol beer [40]. 

From the economic reasons, re-pitching yeast is a common practice within the craft beer production. High yeast vitality, which measures the health of the yeast, is thought to improve fermentation and product quality. The health of the yeast is related to the efficiency and predictability of fermentation as well as the flavour and taste of the final beer. Thus, the vitality of the yeast can directly affect the brewery’s output and financial viability [47]. Correct cropping, storing, and pitching of the yeast are essential for successful fermentation. To avoid microbiological contamination during these processes, precautions should be taken [39]. Additionally, in order to prevent the loss of yeast quality, re-pitching yeast is often restricted to fewer than ten times [48]. Better-maintained yeast will produce more sulfite and less fusel alcohols than older or infected yeast [49]. Long-term re-pitching yeast can lead to degradation due to stress-induced physiological changes, genetic changes to the initial culture, and cross-contamination with other cultures or wild microorganisms [50]. The frequency of yeast pitching affects the taste of the beer as well. Higher pitching rates accelerate the fermentation, but ultimately, they compromise yeast health. 

Microbial contamination, with the main representatives *Staphylococcus*, *Leuconostoc pseudomesenteroides*, and *Acetobacter* sp., also occurs within the bottling room [18]. 

High yeast counts in packed beer or prolonged contact with yeast after fermentation can provide yeasty flavours, which can develop into marmite or meaty flavours as the yeast degrades. If cask or bottle conditioning is employed, yeast counts should be in the range of 0.52 million cells/mL once primary fermentation and the diacetyl rest have been completed. The beer should then be separated from the yeast [39].

The production of flavour compounds (esters and phenolic compounds) has been reported to improve the taste of beer in these mixed cultures of *Pichia kluyveri* and *Brettanomyces*, *Torulaspora delbrueckii* and *Saccharomyces cerevisiae*, *Naumovozyma dairenensis* and *Saccharomyces cerevisiae* [51]. On the other hand, overproduction of esters can give the beer a bitter, overly fruity flavour. Maintaining appropriate conditions is essential for the brewer to establish a balanced organoleptic profile in the beer [52]. Among the most utilised methods meant to identify microbiological spoilage of beer are plate counts, polymerase chain reaction, and flow cytometry [46]. 

## 4. Chemical Safety of Beer

### 4.1. Mycotoxins

Moulds, such as *Aspergillus*, *Penicillium*, and *Fusarium,* produce toxic secondary metabolites called mycotoxins. Almost every mycotoxin is an immunosuppressive agent and can be classified as a hepatotoxin, neurotoxin, nephrotoxin, or carcinogen, having an acute or/and chronic impact on human and animal health [53]. Mycotoxins have been reported in various types of food, but the most important source of these metabolites are globally consumed foods such as cereals (wheat, barley, corn, or rice) [54]. Nowadays, consumers choose high-quality beers with rich and unique sensorial proprieties from small and local, independent breweries. Craft brewers are a growing economic force globally, but the mycotoxin presence has been proven (Table 3) and may impact the quality of the beer [55]. Although barley malt is most often used in brewing and distilling, it is increasingly utilised as a component in several culinary and pharmaceutical products. 

Fungal contamination of barley grains and malt, particularly by *Fusarium* species, can quickly compromise the safety and quality of malt and beer. The contamination of beer by fungi is linked with the “gushing” phenomenon, which causes excessive foaming of the beer leading to overflowing [25]. Other negative effects of barley contamination are reduced malt production productivity, kernel plumpness, and germination [64].

The most common mycotoxins, that have been reported in various barley and malt samples are: nivalenol (NIV), deoxynivalenol (DON), zearalenone (ZEA), deoxynivalenol-3-glucoside (DON3G), fusarenon-X (FUS-X), 3-acetyl-deoxynivalenol (3ADON), 15-acetyl-deoxynivalenol (15ADON), HT-2 toxin (HT-2) and T-2 toxin (T-2) [53]. One of the most found mycotoxins in barley is DON, mainly produced by *Fusarium graminearum* [54].

To illustrate the contamination of worldwide beers with mycotoxins, some relevant studies were discussed in the following paragraphs.

In this way, in the research conducted by Schothorst and Jekel in 2003, fifty-one beers were studied in the Netherlands, and it was found that three beers presented low quantities of DON (ranging from 26 µg/L to 41 µg/L). The white beer from the Netherlands has the highest level of DON (41 µg/L), followed by the white and ale Belgium beer (36 µg/L and 26 µg/L). The beers analysed were not found with trichothecenes above the quantification limit (25 µg/L). In terms of temporary tolerance limit, none of the fifty-one beers exceeded the limit for DON in the Netherlands (500 µg/kg) [56]. 

Moreover, the same idea was presented in 2015 by Piacentini et al., where two relevant mycotoxins were analysed (DON and FB_1_) by HPLC from 53 different Brazilian craft beers (ale and lager). It had no impact on the beer’s physico-chemical properties. In this way, the pH, acidity, and real extract were in agreement with the Brazilian regulation, which means that the level of mycotoxins does not impact the quality of beer. For the ale beer, the following results were obtained: pH (4.55 ± 0.27), acidity (0.26 ± 0.06), and the real extract (5.40 ± 1.48). Similar results were obtained for the lager beer: pH (4.74 ± 0.21), acidity (0.24 ± 0.07), and the real extract (5.17 ± 1.59). From the positive samples, a mean of 221 µg/L (32% from the samples) was registered for the DON and 105 µg/L (15.09% from samples) for the FB_1_. The explication for this level can be due to the toxins in barley being influenced by the environmental conditions (weather, plant growth) and the agricultural methods [25]. 

The same author conducted another study in 2017 on 114 Brazilian lager beers. Of these samples, about 50% have a positive response to FB_1_ ranging between 201.70–1568.62 µg/L. Regarding DON, none of the samples were detected with these mycotoxins. This might be explained because the transmission of the mycotoxins on the final product can depend on the infection of the crop, technological requirements from the brewing process, and agricultural procedures [61].

Another toxic metabolite was evaluated by other authors, namely tenuazonic acid (TA), which is produced by *Alternaria* spp. Forty-three commercial beers were analysed, and it was noticed that the bock beers have the highest average content of TA (37 µg/kg) in comparison with dark, pilsner, alcohol-free, wheat, specialty, or international beer, which contain lower contents of TA (7.4 ± 2.7 µg/kg, 6.8 ± 7.2 µg/kg, 6.3 ± 2.2 µg/kg, 5.6 ± 1.8 µg/kg, and 7.3 ± 3.3 µg/kg, 6.4 ± 7.9 µg/kg) [58]. This variation may appear because of the content of the raw material and top fermentation style, but the data are still limited on this topic. For example, the wheat beer was noticed to have the lowest value for TA. The raw material might explain it because they used wheat instead of barley.

A recent study showed a 26.2% of positive responses to the mycotoxins from 61 Mexican market beers (ale, lager, alcohol-free, 4–5% vol., >5.5% vol., golden, amber, dark-coloured, industrial, craft), but none of them exceeded the limit of quantification of the method used. Of these positive samples, 87.5% were contaminated with DON and its metabolites (DON3G, 3ADON, 15ADON). The high solubility in water of DON, which can be transferred from the malt to the beer, can be the reason for this response. Three beers were contaminated with FB_1_, which means that corn used as an unmalted adjunct could result from contamination. The craft beers also have a higher value for contamination (56.3%) compared with industrial beers (15.55%) [63]. 

This result is also confirmed by Peters et al., 2017, who analysed a selection of 1000 beers (60% craft beers) from 47 countries for the presence of different mycotoxins: aflatoxin B1, ochratoxin A (OTA), fumonisins (FBs), DON, ZEN, T-2, and HT-2 toxins. This study detected more mycotoxins in craft beers than in industrial [53]. Regarding the type of fermentation, in the same study from 2019, it was observed that the ale beers have a higher level of contamination (42%) compared to lager beers (29%), which can be due to the toxin’s adsorption to the yeast cell during the fermentation [53,63].

Moreover, in a study conducted in 2011, 70 artisanal African sorghum beers (*Bil-bil*) were analysed. DON was present in all the samples, and for FB_1_, an incidence of 78.5% was found. On the other hand, for 50 artisanal African sorghum + corn beers (*Kpata*), the incidence of DON was 74% and FB_1_ was 100%. This indicates that the presence of the moulds is due to the natural conditions for African beer production, which includes high moisture values for storage. Furthermore, many mycotoxins can survive in African beer production in different stages (malting, mashing, boiling, fermentation). In terms of tolerable daily intake, the European Commission establishes a temporary value of 1 mg/kg body weight per day for DON and 2 to 4 ppm for FB_1_. For example, for somebody who weighs 60 kg, it means 60 mg of DON/day. If we consider a bottle of 500 mL of beer, we will have a maximum dose of 730 µg/L in *Bil-bil*, meaning 15 bottles per day, which is pretty rare for somebody to consume that amount of beer to reach the maximum value of mycotoxins [59].

Another study was conducted on 83 samples of Italian beer (craft and industrial) using HPLC-FLD for OTA detection, GC–MS for DON, and LC–MS/MS for sterigmatocystin (STC). Low concentrations of the mentioned mycotoxins were reported for both craft and industrial beers, and the values found should not impact the customer’s health [65].

Contrary, it was noticed that the DON level might also decrease, not only increase in the malted barley. For example, a reduction in T-2, HT-2, and some glucosides during processing was observed. This phenomenon may be possible because of the de novo growth of *Fusarium* fungal contaminants. Mould development can be inhibited by certain lactic acid bacteria (LAB), eliminating the toxins. For example, *Lactobacillus plantarum* may act the role of an antifungal compound in malting and brewing processes. Still, more research is required for the antifungal properties of LAB [64].

Similarly, a study conducted by Mastanjević et al., 2018, revealed that *Fusarium culmorum* is not influencing the brewing process negatively because yeasts are fermentative microorganisms and they can adsorb mycotoxins on their cell wall components [66]. The tryptophol concentrations are three times higher in beer samples infected with *Fusarium culmorum*. Tryptophol is a wanted compound in the fermentation industry because it improves flavours and aromas in fermented foods and beverages [66]. 

All the studies showed that a very high level of contamination with the mycotoxins was not present. DON, ZEA, FBs, HT-2, and T-2 seem to be the most studied mycotoxins in barley and beer. Steeping, kilning, mashing, fermentation, and clarification can have an inhibitory effect on the levels of mycotoxins because maybe they are removed, diluted, or destroyed after the thermal treatment. However, it is challenging to remove DON during the technological process because this compound is chemically stable and heat-resistant. On the other hand, ZEN is removed about 60% with spent grains. Some strategies to prevent contamination with the mycotoxins during the process can be applied. For instance, by adding a LAB culture starter during the malting and brewing process, fungicide spread on the field, some yeast strains can bind mycotoxins, etc. [11,29,67]. 

### 4.2. Heavy Metals

Both natural and anthropogenic factors introduce heavy metals into the environment. Metal mining, smelters, shredder plants, trash dumping, and incineration, among other human activities, have raised the concentration of metals in the natural environment, causing their mobilisation to surpass their natural concentration [68].

The discharge of heavy metals into the environment, primarily due to human activity, is a significant source of pollution across the world. Heavy metals, unlike organic contaminants, do not dissolve and remain in the environment permanently, posing a new type of cleanup issue [69]. The number of harmful materials in the environment has risen dramatically as companies have expanded. In addition, greater usage of chemical fertilisers and pesticides can raise heavy metal concentrations in soil and plants. Because of the possible health hazards, the accumulation of heavy metals in soil and plants has become a major issue [68]. 

Some heavy metals have nutritional roles and are required to keep the human body’s metabolism running smoothly. Metal ions such as Cu, Mn, and Zn are required for physiological functions, and some enzymes require metal ions for catalytic activity [68,70]. Other metals, such as Al, Cd, Hg, and Pb, are hazardous even at low quantities and are non-essential for biological processes [68]. 

Heavy metals can be found in beer due to agricultural herbicides, fungicides, and bactericides that contain these harmful metals [67,71]. Rejection of specific contaminated batches, or de-metallisation, is the main action meant to reduce or eliminate this hazard [72]. In their study, Cejka et al. discuss how minerals, whose total presence in the dry matter is roughly 2–3%, play an important role in forming malt extract. On the other hand, trace components make up just around 0.02% of malt extract. This also holds true for hazardous metals, which are transferred from source ingredients to finished beer and brewing residues. Metal concentrations in intermediate phases, such as sweet wort and hopped wort, are determined by their quantity in the raw ingredients (malt, hops, and water) and their ability to transfer into solution during the brewing process [67]. 

Metals in beer are a major source of worry since beer is one of the most popular beverages, and regular use can contribute to the development of new illnesses or exacerbate existing ones [73]. Numerous studies have demonstrated that beer is a rich source of metals that undergo content changes during the process [68,74,75,76]. 

Brewers and customers are particularly interested in determining the overall metal composition of beer, including major, minor, and trace metals [71]. As Eticha, Hymete, and Soares mentioned, metals can be vital or poisonous and can also impact the brewing process and beer quality in terms of flavour stability and haze [68]. Trace metals in beers might be inherent elements of the raw materials, residues of phytosanitary treatments, or contamination from the environment and/or the manufacturing process (processing equipment and containers) [71].

One of the main metals that is mostly discussed is aluminium due to the way it is metabolised in the body because it is often a cause of many major illnesses; the amount of aluminium absorbed may vary. Blanco mentions that it has been reported to increase dramatically in people with Alzheimer’s disease and Down Syndrome (DS) [73]. However, the following factors influence Al content in beers: the brand and chemical content of raw materials, adjuncts, and water used in the manufacturing process; the employment of Al-made processing equipment; the kind and quality of packaging (i.e., purity and type of Al used in can manufacture, type, and quality of the protective lacquer layer); the length of time the can is in contact with the beer (storage period); the pH of the beer, its temperature, and the presence of any harmful substances (such as acids and salts) [77]. Although tin is widely used, beer contamination from raw ingredients, particularly barley and hops, is not. Plants contain just a trace quantity of this metal. Storing raw materials or intermediates in cans, such as must concentrate, can raise the tin concentration [78]. 

Another source of heavy metals in beer is the contamination of brewing equipment, such as pipelines, tanks, containers, and filtration equipment used for handling beer, including fermentation, conditioning, filtration, carbonation, and packaging. Beer contamination can also come from the containers where the completed product is stored and delivered (barrels, aluminium cans) [79].

According to Eticha and Hymete’s 2014 study, the mean concentrations of metals in locally manufactured beer in Ethiopia were as follows: 0.0014 mg/L for Cd, 0.0368 mg/L for Cu, 0.0954 mg/L for Mn, 0.006 mg/L for Pb, and 1.5206 mg/L for Zn. However, the risk evaluation of mean levels revealed no health risk associated with these heavy metals when consumed in beer [68].

Han’s findings talk about how Copper (II) and Lead (II) can be absorbed by waste beer yeast simultaneously. The competing results reveal that while the adsorptive amount for one metal decreases when additional metals become available, the total capacity for binding heavy metals remains relatively constant. Ion exchange was mentioned as one of the primary mechanisms involved in the adsorptive process [80].

Ion exchange, adsorption, chemical precipitation, oxidation, reduction, and reverse osmosis are all ways to treat metal-contaminated wastewater. However, many of these methods are either ineffective or difficult to implement. The high cost of adsorbents, which raises the cost of wastewater treatment, is one of the most significant drawbacks of sorption technologies. Since then, researchers have looked at materials of agricultural and biological origin and industrial by-products as adsorbents in their hunt for a low-cost, readily accessible adsorbent [80].

### 4.3. Biogenic Amines

The quantity of BA in beer is determined by the presence of amino acid decarboxylase activity in many of the microorganisms involved in fermentation. The primary source of biogenic amines in beer is the metabolism of LAB [9]. The most harmful BA for human health is histamine because of its potential allergic or immune response in sensitive individuals. According to Chen and Van Gheluwe (1979), the normal interval histamine can range in beer is 100 to 300 µg/L, depending on the type of beer [81]. The source of BA in beer is most frequently the raw materials (malt) and the contaminating microflora found especially within craft beer production (Table 1). As a result of some stages, elimination compared to industrial beer, such as filtering and pasteurisation, can diminish the shelf life of beer and cause contamination. The genera *Lactobacillus* has a significant effect on the levels of BA, especially in the beers with long-drawn storage time [82]. Dilution of the contaminated batches (mixing with other batches) or withdrawal from production are other actions for reducing the BA level. 

## 5. Physical Contaminants in Beer

It is considered that beer is one of the most contaminated products with micro-plastics [83]. Anthropogenic debris was found in all 12 beer brands processed with municipality water from Laurentian Great Lakes, US (up to 14.3 particles/L). US beer producers tend to filter the beers more thoroughly to increase the beer’s shelf life, which might diminish the micro-plastic contamination. Contrarily, the US microbreweries might remove this step as being considered that which affects the products’ sensory experience, which can explain the higher amount of anthropogenic debris in craft beers [83]. Moreover, in Italy, microfiltration is not allowed in craft beer processing [2]. Higher amounts of micro-plastic contamination were reported in German beers of 2 to 79 fibres/L, from 12 to 109 fragments/L, and from 2 to 66 granules/L, respectively [16]. They pointed out that the potential micro-plastic sources might be materials used in the production process, airborne atmospheric particles, improper cleaning procedures of the beer packaging materials, and even particles found in cereals and hops or other beer ingredients. Still, as water is quantitative, the most important beer ingredient might also be the main beer micro-plastic contaminant [84]. Micro-plastic contamination is strongly linked to improper waste management in urban areas [85], but it also is caused in intensively industrialised areas [86] and even in developed countries’ freshwater [87,88].

## 6. Risk Assessment and the HACCP (Hazard Analysis and Critical Control Points) System in Beer Production

At several phases of the production process, biological, chemical, and physical contaminants can taint beer (Table 4). The HACCP programme is a preventive, methodical approach to beer safety that addresses the risk through prevention. By adopting the HACCP approach, the operators can reduce this likelihood.

The majority of nations throughout the world now apply HACCP as its significance and acceptance grow constantly. The Council Directives 91/43/93 and 92/5/92, in particular, introduced the adoption of HACCP throughout the EU. When additional supplementary quality assurance systems (ISO 9001/2) are already in place, HACCP deployment is much easier. Integrating HACCP and ISO 9001 or ISO 9002 within the context of Total Quality Management is the current trend [96,98,99]. 

A corporation must make an effort to create a HACCP plan before starting a HACCP system, which is typically summarised by the five phases [100,101]: (i) identify HACCP resources and gather the team; (ii) describe the food and its distribution mechanism; (iii) clearly indicate intended use and consumers; (iv) create a process flow diagram, and (v) confirm the accuracy of this diagram in actual use (operation).

HACCP should be viewed as predicated on the legal requirements for Sanitation Standard Operating Procedures in conjunction with Good Manufacturing Practices. The seven HACCP principles listed below serve as the foundation for writing a HACCP [100,102].

1. Perform a risk analysis.
2. Use the HACCP decision tree to find the critical control points (CCPs).
3. Assign each CCP critical limits (CLs).
4. Create a monitoring programme.
5. Create corrective measures.
6. Create a system for keeping records.
7. Create verification protocols.

**Figure 3:**
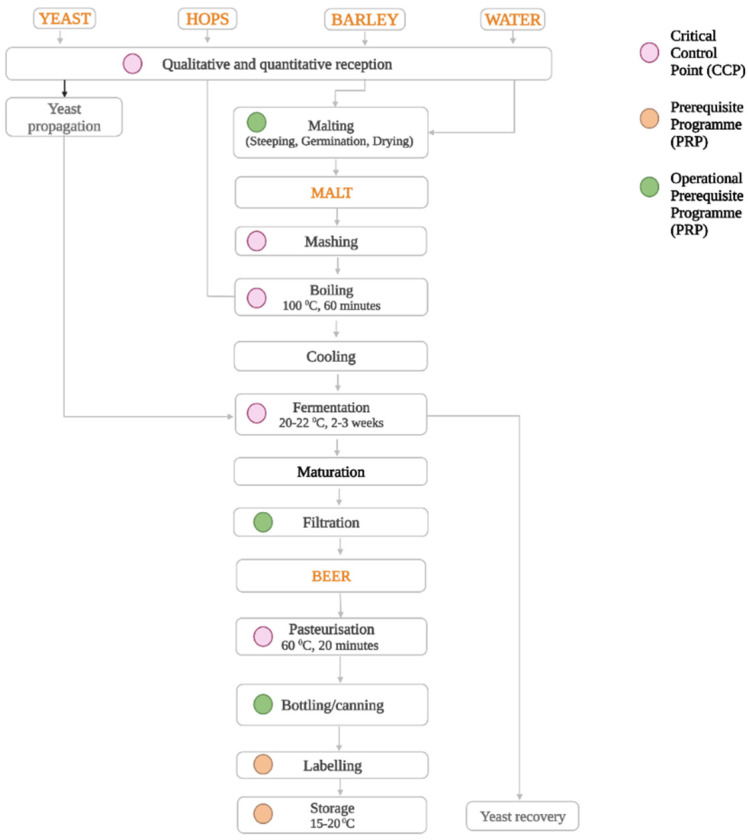
General beer production flow with Critical Control Points (CCPs), Prerequisite Programme (PRP), and Operational Prerequisite Programme (OPRP).

Barley intake, cleaning and grading, drying, steeping, germination and kilning, roasting, milling, mashing, boiling, fermenting, maturing, filtration, and bottling are the main steps in beer production. Figure 3 presents a CCP in a general beer production flow that may jeopardize beer safety.

## 7. Conclusions and Future Trends

There is an increasing concern regarding the impact of human nutrition and safety on the whole life chain. There is a logical concern regarding food contamination and its implications for human health. Since there is a broad list of contaminants and an even broader list of food product types, testing all items designated for consumption is impossible. However, understanding all implications of these contaminants in the food processing system and further in the human body is necessary.

Food risk management and assessment rely on scientific data, and therefore, we reviewed publications on the presence of specific contaminants in beer types. We conclude that the incidence of beer contamination is not to be neglected, identifying a need for specific regulation and standardised analytical determination methods for all identified contaminants. We consider that more clinical studies are needed to help us fully understand the health and environmental implications, also analysing the economic impact (food production, health system expenditures, etc.) of physical, chemical, and biological contaminants in beer. Similarly, the synergic effect of some contaminants or contaminant ingredients (i.e., biogenic amines and ethanol) needs to be deeply addressed. On the other side, because beer is considered an improper medium mostly for biological growth and because the technological parameters sustain biodetoxification, the chemical contamination of beer seems to be the riskiest. Last but not least, legislations regarding the maximum tolerable level of contaminants in beer vary by country, but all authorities have in the spotlight human health and the raw materials (mainly grains) and beer producers. 

## Figures and Tables

**Table 1:** Contamination agents reported in beer produced both craft and large-scale.

Brewing Style	Specificity	Nutritional Aspects	Contaminants Found/Beer-Producing Region	Processing Scale	References
Red	Ale; Dark red, reddish colour, toasty malt flavour, light fruitiness	4.4–6.1% ABV	BA: 12.7–15.5 mg/L (Germany)	NS	[14]
Dark (Stout, Porter)	Ale; Dark brown to black colour	4.4–12.0% ABV	BA: 23.47–30.69 mg/L (Belgium); 10.8–17.2 mg/L (Ireland); 28.2 mg/L (Brazil)	Craft NS	[15]
Pale	Ale; Low carbonation, bitter, malty, dry hop flavour	4.6–6.2% ABV	BA: 13.71 mg/L (Belgium); 35.8 mg/L (Spain); 20.35 mg/L (Spain); 6.2–19.0 mg/L (Belgium)	Craft	[8,15,16]
Wheat wine	Ale; Gold to black colour, bread, honey, caramel/malt aromas, high residual malt sweetness	2.8–12.2% ABV	Micro-plastics: 8–70 n pieces/L fibres 10–50 n/L fragments 8–66 n/L granules (Germany) BA: 7.2–13.1 mg/L (Spain)	Craft	[16]
Fruit	Ale/Lager; Pale to dark colour, malt and hop aromas medium-low perceived	2.5–12% ABV	BA: 14.8–74.1 mg/L (Europe)	NS	[17]
Gueuze	Ale; Gold to medium amber colour, hop aroma is not present to very low, cheesy, floral, or lavender-like attributes	5.0–8.9% ABV	BA: 5–163.1 mg/L (Europe)	NS	[17]
Dark	Lager; Rich, malt sweetness, hints of chocolate, caramel, low hop bitterness	4.5–5.6% ABV	BA: 15.54–78.90 mg/L (Spain)	NS	[15]
Bock	Lager; Intense malt aroma, toasty overtones, rich maltiness, no hop flavour	6.3–7.2% ABV	BA: 23.4 mg/L (Brazil)	NS	[8]
Pale	Lager; Straw to gold colour, medium low to medium perceived malt and hop aromas	5.6–7.0% ABV	BA: 7.03–29.05 mg/L (Spain); 18.56 mg/L (Portugal)	NS	[15]
Pilsner	Lager; Medium-low to medium hop and malt aroma and flavour	4.9–6% ABV	Micro-plastics: 2–79 n-pieces/L fibres; 6–88 n/L fragments; 2–61 n/L granules (Germany) BA: 11.73 mg/L (China);	Craft Large-scale	[17,18,19]

ABV—Alcohol by volume; BA—biogenic amines; NS—not specified.

**Table 2:** Different types of beer spoilage by different bacteria.

Beer Type	Contaminant	Contamination Level	Determination Method	Impact on Beer Quality	References
**Lager**	*P. damnosus*	no beer-spoilage potential	mass spectrometry (MALDI-TOF MS)	Sour beer: diacetyl and lactic acid production; decreased foam stability and sediments could also be the result; major *Pediococcus* infections were referred to as sarcina sickness	[31,40]
	*L. backii*	strong beer-spoilage potential		acidified beer	
	*L. paracollinoides*	strong beer-spoilage potential			
	*L. lindneri*	middle beer-spoilage potential			
	*L. brevis*	strong beer-spoilage potential			
	*Pediococcus claussenii*	1.5 × 10^6^ CFU/mL	RT-qPCR analysis	unfavourable sensory profile	[34]
**Craft**	*L. brevis*	1.16 × 10^2^ CFU/mL	plate culture method with catalase	diacetyl and ropiness, which cause turbidity and beer sourness through the development of lactic and acetic acids	[40,41]
	*L. plantarum*	1.01 × 10^2^ CFU/mL			
	*L. acetotolerans*	8.2 × 10 CFU/mL			
	*P. damnosus*	10^2^ CFU/mL			
**Draft**	*Staphylococcus xylosus*	strong beer-spoilage potential	Advanced beer detection agar plate	beer turbidity organic acids and biogenic amines	[38]
	*B. cereus*	strong beer-spoilage potential	plate culture method	beer turbidity changes in beer flavour	[42]

RT-qPCR = Reverse Transcription - Quantitative Polymerase Chain Reaction

**Table 3:** Occurrence of detected mycotoxins in beers worldwide.

Beer Type	Contaminant	Contamination Level/Limit of Detection (Mean) (µg/L)	Determination Method	Impact on Beer Quality	References
White beer	DON	41	GC–FID	Has not been tested	[56]
White beer		36			
Ale beer		26			
Tusker lager beer	DON	3.29	ELISA	Has not been tested	[57]
	FB_1_	0.28			
	ZEA	0.00784			
Pilsner lager beer	DON	3.57			
	FB_1_	0.32			
	ZEA	0.0085			
Bock beer	TA	37 µg/kg	HPLC–ESI ion-trap multistage MS	Has not been tested	[58]
Artisanal African sorghum beer (*Bil-bil*)	DON	450	ELISA	Has not been tested	[59]
	FB_1_	150			
Artisanal African sorghum beer + corn (*Kpata*)	DON	520			
	FB_1_	210			
Brazilian craft beer (ale and lager)	DON	221	HPLC	pH, acidity, and real extract in agreement with the Brazilian regulation	[25]
	FB_1_	105			
German and imported beer (wheat, barley, or rye)	DON	2.1	EIA	Has not been tested	[60]
	AOH	0.18			
	ZEA	0.14			
	Ergometrine	0.06			
Brazilian market beer (lager)	FB_1_	367.47	HPLC	Has not been tested	[61]
Small-scale brewed beer (regular, wheat, double malt, dark)	OTA	0.005	HPLC-FLD	Has not been tested	[62]
	DON	11.3	GC–MS		
	STC	0.002	LC–MS/MS		
Large-scale brewed beer (regular, wheat, double malt)	OTA	0.008	HPLC-FLD		
	DON	5.8	GC–MS		
	STC	0.001	LC–MS/MS		
Mexican market beers (ale, lager, alcohol-free, 4–5% vol., >5.5% vol., golden, amber, dark-coloured, industrial, craft)	DON	51.76	UHPLC–MS/MS	Has not been tested	[63]
	DON3G	22.36			
	3ADON	4.97			
	15ADON	2.65			
	ZEN	14.12			
	FB_1_	42.77			

AOH = Alternariol; DON = deoxynivalenol; EIA = enzyme immunoassays; ELISA = enzyme-linked immunosorbent assays; FB_1_ = fumonisin B1; GC–FID = gas chromatography–flame ionisation detection; GC–MS = gas chromatography–mass spectrometry; HPLC–ESI ion-trap multistage MS = high-performance liquid chromatography/electrospray ionisation ion-trap multistage mass spectrometry; HPLC-FLD = high-performance liquid chromatography with fluorescence detection; LC–MS/MS = liquid chromatography–mass spectrometry; OTA = ochratoxin A; STC = sterigmatocystin; TA = tenuazonic acid; UHPLC–MS/MS = ultra-high performance liquid-chromatography–mass spectrometry; ZEA = zearalenon; DON3G = deoxynivalenol-3-glucoside; 3ADON = 3-acetyldeoxynivalenol; 15ADON = 15-acetyl-deoxynivalenol.

**Table 4:** General overview of the technological flow, hazards, and critical control points, for beer production.

Stage in the Technological Flow	Description	Critical Control Point (CCP)
Reception of raw and auxiliary materials	The four main ingredients needed to make beer are water, malted barley, hops, and yeast [39]. For barley to germinate and deliver a decent amount of output, it must be of adequate good malting quality. A substantial risk to human health exists when heavy metal concentrations exceed the legislation and when mycotoxin production exceeds 0.04 mg/L, mostly from *Fusarium* species, including aflatoxins, ochratoxin A, zearalenone, and deoxynivalenol [21,68,72,89]. The selection, upkeep, and provision of suitable resources (i.e., strains, pure water), as well as routine assessments of purity and the detection of microbial contamination, make up the quality control of raw materials [9,38,58,81,82].	YES
Malting	The malting process entails soaking the barley in a thin layer of water at a certain temperature to increase its moisture. There could be physical, chemical, or biological risks in this process. A unique chemical hazard with a critical limit (CL) at 0.5 ppb is the generation of nitrosodimethylamine (NDMA) [90]. Furthermore, the production of mycotoxin at a level greater than 0.004 mg/L and changes in flavour and colour pose chemical and physical risks [26,30].	NO, it is an OPRP
Milling	Milling the malt particles facilitates the extraction of endosperm’s soluble components, mostly sugars, and nitrogenous substances. Indirect heating methods and carefully maintained, controlled low-nitrogen oxides burners are also effective ways to reduce the amount of NDMA in malt [72,90].	NO
Mashing	In the initial phase of making wort, called mashing, soluble components of the milled malt are extracted. Production of NDMA (CL = 2.5 ppb) may pose chemical risks to the general public’s health [15,78,91]. Continuous processing monitoring and needed corrections are appropriate preventative and corrective measures that may help keep this CCP under control.	NO, it is an OPRP
Lautering	The wort is fed to the kettle after being circulated through the lauter tun until it achieves a specified level of clarity. Production of apparent total N-nitroso compounds during lautering that is higher than the CL of 20 ppb is considered an OPRP and should be tracked by chemical and microbiological investigations [15,20,92].	NO, it is an OPRP
Boiling	Enterobacteriaceae from hops can cause wort contamination, which can lead to a variety of bad flavours, including “vegetable” and “phenolic” taints tastes [23,24,42,93]. After adding hops, the wort is heated to the boiling point at atmospheric pressure for up to two hours. Wort boiling leads to (i) wort sterilisation and enzyme inactivation; (ii) extraction of bitter and other compounds from the hops and creation of flavour compounds; (iii) wort concentration and evaporation of unfavourable volatile flavours.	YES
Clarification	Either filtering or sedimentation is used to clarify worts.	NO
Cooling	The clear hopped wort is cooled, typically in a plate heat exchanger, in order to get it ready for fermentation. It is advised to aerate or even oxygenate the wort while it is cooling.	NO
Fermentation	While wide different varieties of ales are made by top fermentation (*Saccharomyces cerevisiae*, 18–22 °C), several lagers are produced by bottom fermentation (*Saccharomyces uvarum*, 7–15 °C). At this step, ethanol is produced. Some yeast strains have a propensity to flocculate, trap CO_2_, and rise to the top, whereas others do not flocculate and precipitate. The risk identified at this point is microbial contamination with lactic acid bacteria belonging to *Lactobacilli* and *Pediococcus* (identified by plate count, flow cytometry, and polymerase chain reaction), which generate taints during maturation or in bottle storage [18,19,21,39,46].	YES
Maturation	Lagers are stored at substantially colder temperatures than ale, which matures at relatively mild temps of 12 to 20 °C and involves changes that take place between the end of primary filtration and fermentation.	NO
Filtration	Beer that has been fermenting should be clarified because it is cloudy. Yeasts and proteinaceous components connected to carbs and polyphenols are the cause of this turbidity. It is believed that the chilly temperature, low pH, and poor solubility in alcoholic solutions are to blame for the production of these protein precipitates [89,94]. Dissolved oxygen with a control limit of 0.2 ppm should be used since oxygen uptake during this procedure could significantly impact the product’s organoleptic properties [95].	NO, it is an OPRP
Pasteurisation	Beer undergoes pasteurisation to extend its shelf life over several months. This involves exposing the beer bottle to 60 °C for 20 min. A possible physical hazard is over-pasteurisation, which results in oxidation that negatively influences beer flavour [72,96]. Pasteurisation time and temperature are crucial to be monitored.	YES
Bottling/canning	The bottles/cans cleansing and disinfection are crucial, and the filler line and the sealer are among the OPRPs that make up the packaging section. Possible OPRP includes inadequate cleaning of reusable bottles caused by low temperatures and concentrations of the cleaning solutions, as well as the presence of unwanted materials trapped inside bottles. Additionally, shards and remains of the cleaning solution introduced throughout the procedure create issues. Cleaning and disinfecting agents could contaminate the beer filler [6,97].	NO, it is an OPRP
Labelling	The package’s label should adhere to the legislation and standards for the labelling of prepackaged foods and beverages.	NO, it is a PRP
Bottle/can packaging	Physical risks related to the bottles’ (cans’) condition may be encountered during the procedure.	NO, it is a PRP
Storage	To ensure the final product qualities fall within the acceptable range, chemical, microbiological, and organoleptic analyses are performed.	NO, it is a PRP

CL—Critical Limit; PRP—Prerequisite Programme (a preventative step that the hazard analysis determined was necessary to reduce the risk, focused on hygienic status); OPRP—Operational Prerequisite Programme (crucial to minimising the risk’s likelihood, focused on cross-contamination).

## Data Availability

No new data were created or analyzed in this study. Data sharing is not applicable to this article.
